# Circular RNAs: from transcriptional noise to engineered therapeutics

**DOI:** 10.3389/freae.2026.1894220

**Published:** 2026-09-17

**Authors:** Aaron C. Chack, Chad V. Pecot

**Affiliations:** 1 Lineberger Comprehensive Cancer Center, University of North Carolina at Chapel Hill, Chapel Hill, NC, United States; 2 RNA Discovery Center, University of North Carolina at Chapel Hill, Chapel Hill, NC, United States; 3 Curriculum in Genetics and Molecular Biology, University of North Carolina at Chapel Hill, Chapel Hill, NC, United States; 4 Division of Oncology, University of North Carolina at Chapel Hill, Chapel Hill, NC, United States

**Keywords:** biogenesis, cancer, circular RNAs, dysregulation, non-coding RNAs, therapeutics

## Abstract

Circular RNAs (circRNAs) are single stranded, closed, primarily non-coding RNA circles that have risen to the forefront of the non-coding RNA field. Studies from recent years have shown that circRNAs are produced from endogenous, non-canonical RNA splicing mechanisms, demonstrate specific cellular localizations, regulation by RNA modifications and function in many fundamental and critical cellular processes. CircRNAs can influence gene expression both pre- and post-transcriptionally, influence translation and interact with cellular RNA and proteins to enable cellular homeostasis. Aberrations in circRNA expression patterns lead to alterations in gene regulatory networks, which have been implicated in the progression of many cancer types. Unique characteristics of circRNAs, particularly their stability within cells and ability to be leveraged for protein production, have led to the engineering of artificial circRNAs to be used as an emerging therapeutic modality. Here we review how circRNAs regulate homeostasis in normal tissues, how dysregulated expression of circRNAs impacts various cancers and emerging therapeutic technologies that build upon their unique features.

## 1 Introduction

Over the last half century, RNA has proved to be much more than just a message for proteins. As less than 6% of the transcriptome encodes protein, the overwhelming majority of RNA is categorized as non-coding RNA (Consortium, 2012). CircRNAs are a class of primarily non-coding RNAs that consist of a covalently closed, single stranded circle. RNA was first observed in a circular configuration in the 1970s by electron microscopy, first in pathogenic plant viroids (Sanger et al., 1976), and later from virions and RNA virus genomes in eukaryotic cells (Hewlett et al., 1977; Hsu and Coca-Prados, 1979). These molecules are not included in today’s definition of circRNAs as they are generated via rolling circle replication or not covalently linked. Decades later, eukaryotic transcripts were discovered that corresponded to “scrambled exons” and were thought to be stable, circular, mis-spliced products of normal messenger RNA (mRNA) splicing as the observed circRNA expression was much lower than that of their linear RNA counterparts (Capel et al., 1993; Cocquerelle et al., 1992; Cocquerelle et al., 1993; Nigro et al., 1991). Lastly, intentional changes in RNA sequencing preparation and analysis revealed that circRNAs are transcribed from many human genes, conserved across species and possess expression levels comparable or greater than linear RNAs from the same locus (Jeck et al., 2013; Salzman et al., 2012). A subsequent study demonstrated circRNA expression in eukaryotes more broadly (including fungi and single-cell protists), suggesting a conserved mechanism of gene expression (Wang et al., 2014).

In the years since, endogenous circRNAs have been shown to be players in many fundamental cellular processes. CircRNAs can regulate expression of RNA, mRNA translation, influence transcription at individual loci, induce RNA degradation and interact with a multitude of RNA binding proteins (Chen, 2016; Das et al., 2021). These functions are generally enacted through direct binding of a circRNA to other small cellular RNAs; while this direct binding to other RNAs is an important mechanism, it is not universal. Moreover, specific RNA secondary structures of circRNAs function in the innate immune response to foreign double-stranded RNA (Liu et al., 2019). CircRNAs are crucial for maintaining cellular homeostasis, while aberrant expression of circRNAs may lead to several disease states, including cancer initiation and progression (Conn et al., 2024; Kristensen et al., 2022). In addition to being key biologically active molecules, unique characteristics of circRNAs are being exploited to design and develop improved platforms for delivery of therapeutic agents to improve human health.

In this review, we summarize the different circRNA biogenesis pathways and normal cellular functions, followed by descriptions of aberrant circRNA expression patterns and cellular functions in cancer. We discuss the involvement of RNA modifications, specifically N(6)-methyladenosine (m6A), on circRNA biogenesis, function and cellular mechanisms in cancer. We conclude by describing current advances in engineering circRNAs as a therapeutic modality. In-depth reviews detailing techniques used to identify circRNAs, their biogenesis and normal physiologic functions have been published extensively elsewhere (Chen, 2016; Jeck and Sharpless, 2014; Kristensen et al., 2019; Liu and Chen, 2022). The goal of this review is to offer an overview of the current circRNA field, from the basics of normal circRNA biology and epigenetic regulation to the advancements in engineering circRNAs to treat disease. This review will highlight the importance of alternative circRNA functional mechanisms, as well as discuss challenges, criticisms and suggest new experimental directions as the circRNA field continues to grow and evolve. Studies referenced in this review were selected based on in depth experimental validation, relevance to cancer models, novel proposed functions of circRNAs or a focus on understudied circRNA classes.

## 2 Biogenesis and subclasses of circRNAs

The predominant pathway of circRNA biogenesis is through a process known as backsplicing. Backsplicing is a non-canonical form of alternative splicing by which an upstream splice acceptor site is joined with a downstream splice donor site to produce a covalently closed RNA loop (Figure 1A). The two splice sites are often brought into close proximity by RNA-RNA interactions of inverted, short, repetitive sequences within the flanking introns, such as Alu elements or mammalian-wide interspersed repeats (Ivanov et al., 2015; Jeck et al., 2013; Liang and Wilusz, 2014; Yoshimoto et al., 2020). The endogenous pre-mRNA spliceosome facilitates ligation of the circRNA backsplice junction as the RNA is being transcribed (Ashwal-Fluss et al., 2014). Circularization and canonical, linear mRNA splicing compete with one another, and depletion of spliceosomal proteins promotes backsplicing events (Ashwal-Fluss et al., 2014; Liang et al., 2017). In addition to inverted intronic repeats, several RNA binding proteins (RBPs) have been shown to facilitate backsplicing. For example, the RBP MBL binds to the introns flanking the second exon of its own gene and promotes generation of circMbl in mouse neuronal cells (Ashwal-Fluss et al., 2014). Likewise, RBPs FUS and QKI bind their respective binding sites in flanking introns to drive the biogenesis of multiple circRNAs in neuronal cells and during epithelial to mesenchymal transition in human mammary epithelial cells, respectively (Conn et al., 2015; Errichelli et al., 2017). Though the overall backsplicing process is similar, the endogenous intronic sequences and involved RBPs/splicing machinery can vary extensively for each individual circRNA and be tissue type or cellular context dependent.

**FIGURE 1:**
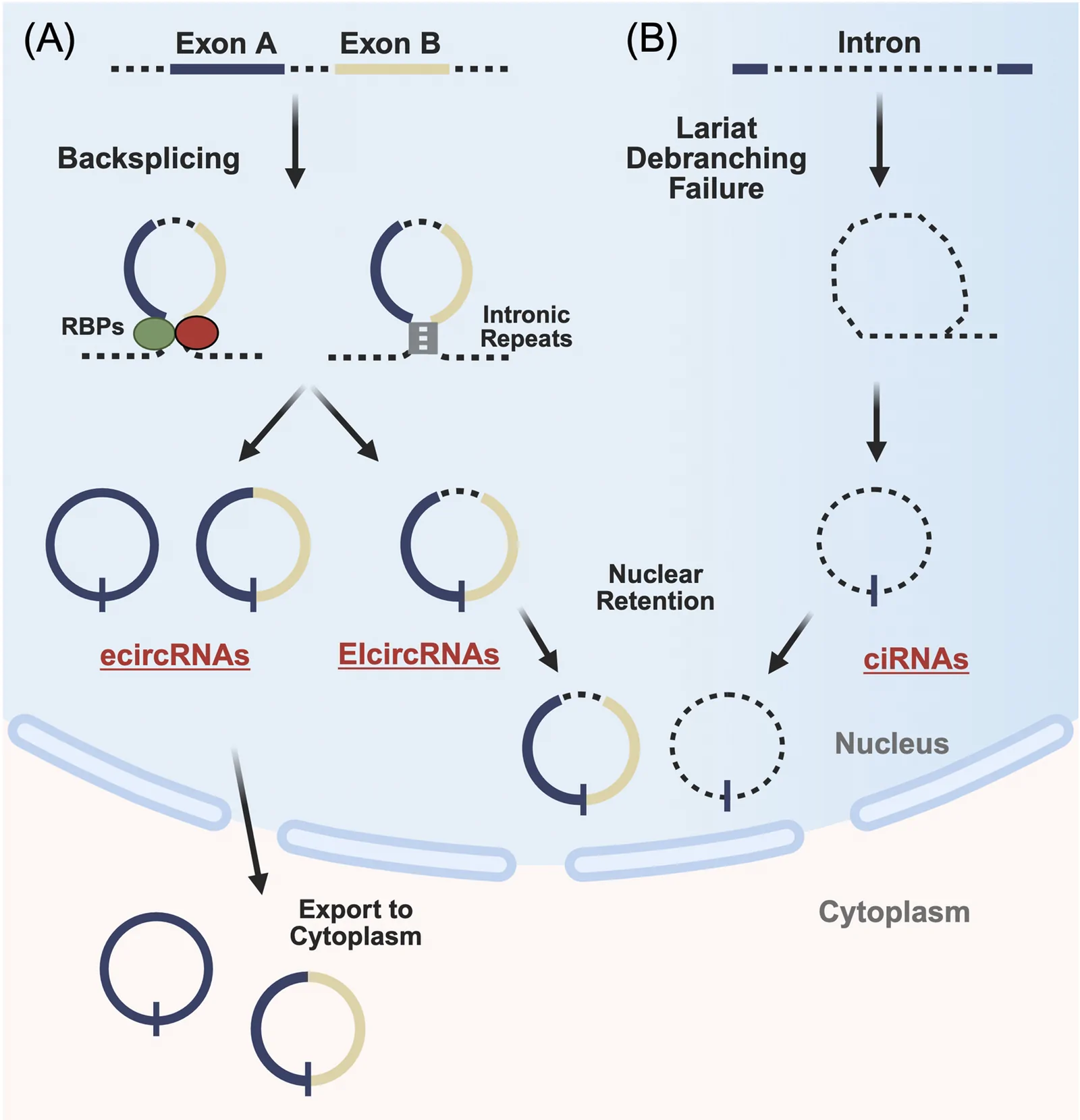
Common mechanisms of circRNA biogenesis. **(A)** Simplified schematic of backsplicing. 5′ and 3′ exon ends are brought into close proximity by RBPs bound to the intron or direct interactions of complementary repeats in the intron. Backsplicing gives rise to ecircRNAs and EIcircRNAs. **(B)** Simplified schematic of lariat debranching failure, giving rise to ciRNAs (Figure created with BioRender, in part by adapting publicly available templates. Publication License - Created in BioRender. Chack, A (2026) https://BioRender.com/f613q8s).

CircRNAs can be subclassified into three main types based on their sequence composition. Mature circRNAs are generally derived from exonic sequences, intronic sequences, or a mixture of exonic and intronic sequences (Table 1). Additional circRNAs are predicted to derive from intergenic regions, and there is evidence that circRNAs can be generated from fusion genes and the splicing of transfer RNAs (tRNAs) (Gao et al., 2015; Lu et al., 2015; Tan et al., 2018). These require further characterization and do not currently fit into the three main subclasses. Though circRNAs are composed of various sequences of various lengths, they share common features. The lack of free 5′ and 3′ RNA ends prevents 5′ caps or 3′ poly (A) tails and affords extreme stability in cells due to inherent exonuclease resistance. Additionally, the backsplicing process brings the linear 5′ and 3′ ends together to produce a unique splice junction not observed in the cognate linear mRNA, known as the backsplice junction.

**TABLE 1:** Classification, cellular localization and functions of common circRNA types.

Name	Abbreviation	Contents	Cellular localization	Cellular function(s) and references
Exonic circRNAs	ecircRNAs	Exon(s)	Primarily Cytoplasm	ceRNAs (Hansen et al., 2013), mRNA stabilizers (Hansen et al., 2011), protein scaffolds (Du et al., 2016), transcriptional regulators (Conn et al., 2017), translational regulators (Abdelmohsen et al., 2017)
Intronic circRNAs	ciRNAs	Intron(s)	Primarily Nucleus	Transcriptional regulators (Zhang et al., 2013)
Exon-intron circRNAs	EIcircRNAs	Exon(s) and intron(s)	Nucleus	Transcriptional regulators (Li et al., 2015)

### 2.1 Exonic circRNAs

Exonic circRNAs (ecircRNAs) are comprised of one or more exons of a gene, and are the most prevalent and well-characterized subclass of circRNAs. They are generated co-transcriptionally in the nucleus by backsplicing (Figure 1). In addition to standard backsplicing, ecircRNAs can undergo alternative backsplicing, allowing for the production of multiple ecircRNA transcripts from the same genetic locus. While the functional consequences of the alternatively spliced ecircRNAs remain understudied, it seems reasonable to speculate that different isoforms of ecircRNAs would have distinct functions and cellular expression patterns, much like those of mRNAs (Ray et al., 2020). Following successful backsplicing, the majority of mature ecircRNAs are then exported to the cytoplasm. Nuclear export is facilitated by several exportin proteins or complexes, such as the IGF2BP1/Ran-GTP/exportin-2 complex or the DDX39A and DDX39B proteins, the latter of which export ecircRNAs from the nucleus in a size selective fashion (Huang et al., 2018; Ngo et al., 2024). Proper export of ecircRNAs is critical to maintain cellular homeostasis, as enrichment of circRNAs in the nucleus lead to the formation of DNA:ecircRNA hybrids (R-loops) which can induce DNA damage (Chen L. et al., 2022).

Along with being the most abundant subclass of circRNA, ecircRNAs also have the most characterized cellular functions (Table 1). The first ecircRNA to be functionally annotated was cerebellar degeneration-related antigen 1 antisense (CDR1as, also known as ciRS-7). CDR1as was found to stabilize the mRNA transcript of cerebellar degeneration-related antigen 1 (CDR1), which is produced from the opposite strand of CDR1as. CDR1as is also negatively regulated by the microRNA (miR) miR-671 and AGO2 in neuronal tissues (Hansen et al., 2011). Shortly after its discovery, immunoprecipitation and FISH colocalization demonstrated that CDR1as was able to bind miR-7 and reduce degradation of miR-7 mRNA targets. The testis-specific ecircRNA, circSry, was found to have a similar effect with miR-138 (Hansen et al., 2013). Subsequently, the terms “miR sponge” and “competing endogenous RNA” (ceRNA) have been used to refer to circRNAs and other non-coding RNAs that directly interact with miRs, thereby sequestering the miRs from their natural targets. The overwhelming majority of functional studies ascribe the miR ceRNA function to specific ecircRNAs, which have been reviewed in depth previously (Chen, 2016; Kristensen et al., 2019). CircRNAs, their associated miRs and the tools used to predict the circRNA/miR interactions from select studies cited in this review are summarized in Table 2. As an example, the ecircRNA circHIPK3 was demonstrated to sponge nine individual miRs, and disruption of this regulation deters cellular growth (Zheng et al., 2016). The ceRNA description has also been applied to ecircRNAs and other non-coding RNAs that bind and sequester proteins. For example, the RBP IGF2BP2 was found to directly bind to circHIPK3, and depletion of circHIPK3 resulted in the degradation of IGF2BP2 targets (Okholm et al., 2024).

**TABLE 2:** Representative circRNAs, interacting miRs and sources used to identify interacting miRs prior to experimental validation cited in this review.

circRNA	Associated miR(s)	Prediction tool	References
circHIPK3	miR-124, miR-152, miR-193a, miR-29a, miR-29b, miR-338, miR-379, miR-584 miR-654	miR library screen	Zheng et al. (2016)
circHIPK3	miR-558	miRanda, PITA, RNAhybrid	Li et al. (2017)
CDR1as	miR-219a-5p	starBase	Li et al. (2020)
CDR1as	miR-7	MEME	Hansen et al. (2013)
circSry	miR-138	MEME	Hansen et al. (2013)
circMDK	miR-346, miR-874-3p	miRDB, miRanda, TargetScan	Du et al. (2022)
circPLPP4	miR-136	CircInteractome	Li et al. (2024)
circTP63	miR-873-3p	miRanda	Cheng et al. (2019)
hsa_circ_0125356	miR-582-5p	miRanda, TargetScan, CircBank	Du et al. (2025)
circAKT3	miR-198	miRanda, PITA, RNAhybrid	Huang et al. (2019)
circIGF2BP3	miR-328-3p, miR-3173-5p	starBase	Liu et al. (2021)
circHERC1	miR-142-3p	CircInteractome, CSCD, CircBank	Cui Y. et al. (2023)

Apart from acting as ceRNAs for miRs or proteins, ecircRNAs can also bind proteins to form complexes with regulatory effects. For instance, circ-Foxo3 is able to arrest cell cycle entry by binding to and forming a ternary complex with cell cycle regulators CDK2 and p21 (Du et al., 2016). CircPABPN1 associates with the post-transcriptional regulator RBP, HuR, and downregulates translation of its cognate PABPN1 mRNA (Abdelmohsen et al., 2017). In addition to regulating translation of mRNAs, some ecircRNAs harbor open reading frames and can be translated into peptides in a cap-independent manner. Translation of ecircRNAs requires an internal ribosome entry site (IRES) (Legnini et al., 2017; Pamudurti et al., 2017). The cellular function of protein products translated from circRNAs remain understudied, though circZNF609-250aa, the peptide generated from circZNF609, has been shown to be involved in AKT/mTOR signaling in renal cells following injury (Ouyang et al., 2023).

While the majority of ecircRNAs are exported to the cytoplasm, some are retained in the nucleus where they influence transcription. In *Arabidopsis*, nuclear-retained ecircRNAs generated from the SEP3 gene remain at their transcriptional locus where they form R-loops (RNA:DNA hybrids) with the genomic DNA. These ecircRNA-generated R-loops hinder transcriptional elongation and promote backsplicing, constituting a feed forward mechanism by which the ecircRNA enhances its own transcription (Conn et al., 2017).

### 2.2 Intronic circRNAs

Intronic circRNAs (ciRNAs) are formed entirely of intronic sequences and are far less studied and characterized than ecircRNAs. CiRNAs were first identified when RNA sequencing reads (generated with no poly (A) enrichment) mapped to introns. These introns demonstrated no coding potential, were highly abundant and were produced in a cell-type specific manner. These intronic RNAs were subsequently determined to be circular. However, their production did not occur through backsplicing, but through failed debranching of intron lariats formed during normal RNA splicing (yielding a 2′-5′ phosphodiester bond in the RNA) (Figure 1B). Specific GU-rich and C-rich motifs located in the ciRNA sequence (near the 5′ splice site and the intron branch point, respectively) are required for ciRNA formation (Zhang et al., 2013). These ciRNAs are retained within the nucleus where they function in transcriptional regulation, much like the nuclear-retained ecircRNA. The ciRNA ci-ankrd52 accumulates at its parent gene’s transcription start site and directly interacts with Pol II to increase the rate of transcriptional elongation. These interactions allow ci-ankrd52 to increase the transcriptional efficiency of its own gene (Zhang et al., 2013). Furthermore, in rodent pancreatic islet cells, the second intron of the insulin gene *Ins* forms a ciRNA (ci-Ins2). Knockdown of ci-Ins2 in pancreatic islets led to a decrease in insulin secretion, and affected the transcription of genes related to calcium signaling. This change in transcription is thought to be due at least in part to ci-Ins2 binding to the RBP, TDP-43, though several other RBP partners were predicted (Das et al., 2020; Stoll et al., 2020).

Lastly, and contrary to previous work, ciRNAs expressed from a multitude of genes and across species were found to be actively exported into the cytoplasm by the NXF1/NXT1 complex. These exported ciRNAs were not found to associate with other RNAs from their parental gene in the cytoplasm, and any functions they possess remain unknown (Talhouarne and Gall, 2018).

### 2.3 Exon-intron circRNAs

Exon-intron circRNAs (EIcircRNAs) are generated from a combination of exonic and intronic sequences and are the least studied subclass of circRNA. This subclass was first described upon closer examination of circRNAs identified by RNA sequencing. The selected circRNAs harbor both exonic and intronic sequences when analyzed by both RT-PCR and Northern blots (Li et al., 2015). Biogenesis of EIcircRNAs is consistent with backsplicing, though EIcircRNAs are retained in the nucleus, unlike most ecircRNAs also produced by backsplicing (Li et al., 2015) (Figure 1A). It is possible that EIcircRNAs are a product of ecircRNA alternative backsplicing, in which the same locus can give rise to both ecircRNAs and EIcircRNAs.

In the nucleus, EIcircRNAs also function in transcriptional regulation. Knockdown of the EIcircRNAs circEIF3J and circPAIP2 resulted in decreased expression of their parental genes, but had no effect on expression of neighboring genes, suggesting a localized regulation. The EIcircRNAs of interest were shown to directly interact with the promoter regions of their respective parental genes and the U1 small nuclear ribonucleoprotein. The interaction with the U1 small nuclear ribonucleoprotein was necessary for the EIcircRNA influence on parental gene expression, and other EIcircRNAs were found to interact with the U1 small nuclear ribonucleoprotein as well (Li et al., 2015).

### 2.4 Other circRNAs

As introns flanking circRNA sequences contain complementary repetitive sequences or short sequence motifs, several RNA sequencing analysis methods or algorithms have been developed to predict circRNAs. Some of these tools identified potential circRNA sequences in the intergenic regions of the genome (Gao et al., 2015). Another method identified potential circRNAs originating from intergenic regions and from internal regions of exons and introns. Dubbed “interior circRNAs”, some examples have been experimentally validated by RT-PCR and sequencing (Liu et al., 2020). Finally, unique circRNAs have been observed to originate from gene fusions, such as the EML4-ALK gene fusion product in cancer cells and from tRNA intron sequences in several species (Lu et al., 2015; Tan et al., 2018). The functional consequences of these unique circRNAs remain unclear.

In summary, ecircRNAs are overwhelmingly found in the cytoplasm where they impact gene expression through acting as ceRNAs for miRs or RBPs, acting as a binding scaffold for RBPs and by directly or indirectly stabilizing mRNAs. A subset of ecircRNAs harbor IRESs and produce peptide products. CiRNAs are generally retained in the nucleus where they directly and indirectly influence transcription and splicing. Similar to ciRNAs, EIcircRNAs are retained in the nucleus and function through transcriptional regulation. For reasons that are still unclear, the majority of studies focus on ecircRNAs. This may be due to ecircRNAs being more highly expressed than the other subclasses, better annotations of ecircRNAs, or technical challenges involved in identifying and interrogating function of the other subclasses. Further study focusing on transcriptional differences and in-depth identification of members of the circRNA subclasses is required.

## 3 CircRNA expression dysregulation and mechanisms in various cancers

Cancer continues to be the leading cause of death of people under 85 years of age in the United States, and lung cancer is projected to claim more lives in 2026 than the two next most frequent diagnoses combined (Siegel et al., 2026). While localized tumors cause disruptions in their tissue of origin, cancer metastasizing and colonizing distant sites causes the vast majority of cancer deaths (Gupta and Massague, 2006). Tumor profiling by The Cancer Genome Atlas has led to great advances in our understanding of the specific molecular pathways driving various cancers and uncovered actionable targets for new therapies (Cancer Genome Atlas Research et al., 2013). Despite these advances, many cancers still have poor prognoses and the impact of the non-coding RNA transcriptome on the development and progression of cancer remains poorly understood.

Under normal conditions, expression of circRNA varies in both developmental stage and cell type, and appear to be enriched in neuronal tissues. Briefly, distinct subsets of circRNAs were found to be uniquely expressed in either CD19^+^ leukocytes, CD34^+^ leukocytes or neutrophils, and a separate subset of circRNAs are expressed in nematode oocytes but not in later embryos (Memczak et al., 2013). In mice, expression of circRNAs was altered at different developmental stages corresponding to synaptogenesis in the mouse brain (You et al., 2015). Similarly, in the developing rat retina, cellular circRNA levels increased at early postnatal timepoints followed by a decrease at later timepoints (Han et al., 2017). Furthermore, circRNA isoform expression has been found to be unique to certain cell types, with isoform expression changing within the same cell type in a developmental stage-specific manner (Salzman et al., 2013; Veno et al., 2015). Finally, specifically in neuronal tissues, cellular circRNA levels accumulate over time in an age-dependent fashion (Cortes-Lopez et al., 2018; Gruner et al., 2016; Westholm et al., 2014).

An ever-increasing body of evidence points to circRNAs being key players in gene expression regulatory networks in temporal and tissue-specific manners. This tight regulation positions circRNAs to be uniquely susceptible to dysregulation in malignant transformation. Aberrant circRNA expression and the resulting altered regulatory networks can have drastic effects on cellular homeostasis. Accordingly, circRNA expression is frequently altered in cancer where differing levels have been shown to have both oncogenic and tumor suppressive effects, depending on the cancer and specific circRNA. Altered splicing machinery and epigenetic remodeling, transcriptional amplification, and RNA modification pathways (e.g., m6A) in cancer can have marked impacts on circRNA abundance and pathophysiology.

Work in recent years has sought to identify the contributions of circRNAs in cancers and define their related molecular mechanisms. CircRNAs shown to have altered expression and functions in cancer are thus far largely limited to ecircRNAs. Their functions commonly occur through a variety of mechanisms consistent with described circRNA functions under normal conditions (Figure 2). Additional examples not described below and are summarized in Table 3.

**FIGURE 2:**
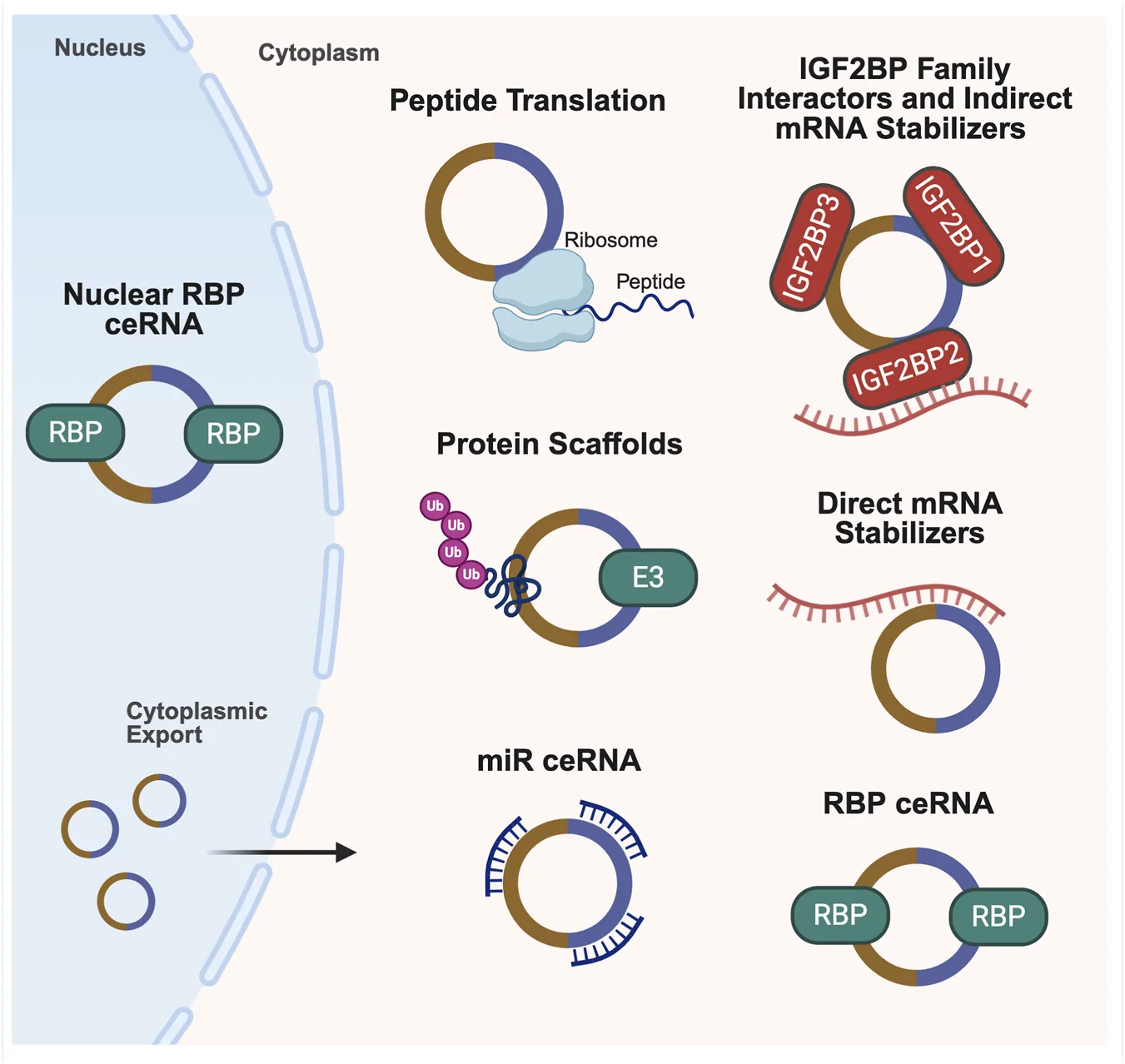
Overview of cellular circRNA functions in cancer. Primarily ecircRNAs have been shown to have functions in cancer. ecircRNAs generally will be exported to the cytoplasm where they function as miR ceRNAs, RBP ceRNAs, protein scaffolds, IGFBP family interactors, stabilize mRNA or are translated. circRNAs may have more than one function, depending on cancer type (Figure created with BioRender, in part by adapting publicly available templates. Publication License - Created in BioRender. Chack, A (2026) https://BioRender.com/z5tc3pq).

**TABLE 3:** Dysregulated circRNAs and identified functions in cancer.

Cancer type	circRNA	Expression*	Function	Consequence	References
NSCLC	CDR1as	Up	miR ceRNA (miR-219a-5p)	Increased SOX5	Li et al. (2020)
NSCLC	CDR1as	Up	mRNA stabilization (CDR1)	Increased CDR1	Harrison et al. (2020)
NSCLC	circ_PPAPDC1A	Up	miR ceRNA (miR-30a-3p)	PI3K/AKT/mTOR pathway activation	Tang et al. (2024)
NSCLC	hsa_circ_0125,356	Up	miR ceRNA (miR-582-5p)	Wnt pathway activation	Du et al. (2025)
NSCLC	circIGF2BP3	Up	miR ceRNA (miR-328-3p and miR-3173-5p)	Tumor PD-L1 stabilization	Liu et al. (2021)
NSCLC	circHERC1	Up	miR ceRNA and protein ceRNA (miR-142-3p and FOXO1	MAPK, NF-KB and AKT pathway activation	Cui Y. et al. (2023)
NSCLC	circNDUFB2	Down	IGF2BP family interactor	Increased IGF2BP family	Li et al. (2021)
NSCLC	circXPO1	Up	IGF2BP family interactor (IGF2BP1)	Wnt pathway activation	Huang et al. (2020)
NSCLC	circFNDC3B	Up	Protein ceRNA (TFII-I)	Decreased T cell infiltration	Wei et al. (2025)
NSCLC	circATF6	Down	Protein ceRNA (BiP/GRP78)	Increased resistance to drug pressure	Hu et al. (2025)
NSCLC	circTP63	Up	miR ceRNA (miR-873-3p)	Increased FOXM1	Cheng et al. (2019)
NSCLC	cLMB1	Down	Protein interactor (FGFR4 and CBL)	FGFR4 degradation	Qian et al. (2025)
GC	circMAPK1	Down	Peptide Translation	MAPK pathway suppression	Jiang et al. (2021)
GC	circNRIP1	Up	miR ceRNA (miR-149–5p)	PI3K/AKT/mTOR pathway activation	Zhang et al. (2019)
GC	circAKT3	Up	miR ceRNA (miR-198)	PI3K/AKT/mTOR pathway activation	Huang et al. (2019)
GC	circ-hnRNPU	Down	Protein ceRNA (NONO)	c-Myc transactivation, protein glycosylation	Li H. et al. (2023)
GC	circPAK2	Up	mRNA stabilization (though IGF2BPs binding)	Increased VEGFA stability	Ding et al. (2024)
HCC	circMDK	Up	miR ceRNA (miR-346 and miR-874-3p)	PI3K/AKT/mTOR pathway activation	Du et al. (2022)
OC	circPLPP4	Up	miR ceRNA (miR-136)	PI3K/AKT/mTOR pathway activation	Li et al. (2024)
BC	FECR1	Up	Protein interactors (TET1 and DNMT1)	Induce promoter hypomethylation	Chen et al. (2018)
BC	circFOXK2	Up	mRNA stabilization	Increased CCND1	Yi et al. (2025)
TNBC	circRNA-CREIT	Down	Protein interactor (PKR and HACE1)	RACK1/MTK1 pathway activation	Wang et al. (2022)
TNBC	circFBXW7	Down	miR ceRNA (miR-197–5p) and Peptide Translation	Increased FBXW7 and suppressed c-Myc	Ye et al. (2019)
CC	circRNF13	Up	mRNA stabilization	Increased radioresistance	Shi et al. (2023)
CC	CDR1as	Up	mRNA stabilization (though IGF2BP1 binding)	Increased SLUG stability	Zhong et al. (2023)
OR	circRNF13	Up	mRNA stabilization (though IGF2BP1 phase separation)	Increased ITGB1 stability	Xu et al. (2025)
M	CDR1as	Down	Protein ceRNA (IGF2BP3)	IGF2BP3 release	Hanniford et al. (2020)
CRC	circNSUN2	Up	mRNA stabilization (though IGF2BP2 binding)	Increased HMGA2 stability	Chen R. X. et al. (2019)
CRC	circITGB6	Up	mRNA stabilization (though IGF2BP3 binding)	Increased PDPN stability and EMT	Li K. et al. (2023)
CRC	circ_0007379	Up	Other (miR processing scaffold)	Decreased RUNX1	Long et al. (2023)
PC	Hsa_circ_0038737	Up	mRNA stabilization (though IGF2BP3 binding)	Increased DNPH1 stability	Wang et al. (2025)
GL	circFBXW7	Down	Peptide Translation	c-Myc destabilization	Yang et al. (2018)
GL	circ-SHPRH	Down	Peptide Translation	SHPRH stabilization	Zhang et al. (2018)
CNC	circFNDC3B	Down	Peptide Translation	Snail suppression	Pan et al. (2020)
CNC	circPPP1R12A	Up	Peptide Translation	Hippo-YAP pathway activation	Zheng et al. (2019)
BLC	circHIPK3	Down	miR ceRNA (miR-558)	Heparanase suppression	Li et al. (2017)

* Expression relative to levels in normal, respective tissue.

NSCLC, Non-small cell lung cancer; GC, Gastric Cancer; HCC, Hepatocellular carcinoma; OC, Ovarian cancer; BC, Breast cancer; M, Melanoma; OR, Oral Cancer; CRC, Colorectal Cancer; PC, Prostate Cancer; CC, Cervical Cancer; TNBC, Triple Negative Breast Cancer; GL, Glioma; CNC, Colon Cancer; BLC, Bladder Cancer.

### 3.1 miR ceRNAs

Consistent with reports of circRNAs under normal conditions, the most frequently reported function of circRNAs in cancers is acting as a ceRNA for a wide variety of miRs. Notably, many oncogenic circRNAs converge on common downstream signaling pathways, including PI3K/AKT/mTOR, Wnt signaling, and mechanisms that promote immune evasion. The most frequently described miR ceRNA function for circRNAs in cancer involves upregulation of an “oncogenic” circRNA which is thought to induce binding and sequestering of associated tumor suppressive miRs. This sequestering results in increased expression of the miR’s target gene(s). Conversely, downregulation of “tumor suppressor” ceRNAs would be anticipated to have the opposite effects. Imbalances in these regulatory networks can lead to increased growth, invasion and metastasis of cancer cells. Increases in circRNA generally correlate with worse prognosis and/or stage and circRNA regulatory network dysfunction can also lead to enhanced resistance of various anti-cancer therapies.

Some ceRNAs display increased expression in cancer cells and have been demonstrated to induce activation of the PI3K/AKT/mTOR pathway (Manning and Toker, 2017). For example, in non-small cell lung cancer (NSCLC), up-regulated circ_PPAPDC1A binds miR-30a-3p, which increases IGFR1 levels and confers resistance to the kinase inhibitor Osimertinib by activating the PI3K/AKT/mTOR pathway. Overexpression of circPPAPDC1A and silencing of circPPAPDC1A led to increased and decreased growth rates, tumor size and tumor weight *in vivo*, respectively (Tang et al., 2024). Moreover, AKT3 produces a circRNA, circAKT3, which is upregulated in gastric cancer cells that are resistant to cisplatin treatment. CircAKT3 was found to directly bind miR-198, which upregulates PIK3R1 and promotes PI3K/AKT/mTOR pathway activation *in vitro*. Silencing of circAKT3 improved response to cisplatin in *in vivo* xenografts, presumably through the same mechanism (Huang et al., 2019). However, phenotypic rescue experiments were not performed in either of these studies. Finally, and as another example of a pathway activation, upregulated hsa_circ_0125356 was found to directly bind to miR-582-5p and act as a ceRNA, which in turn upregulated FGF9 and activated both canonical and non-canonical Wnt signaling pathways, leading to enhanced gemcitabine resistance in NSCLC *in vivo* (Du et al., 2025). Several circRNAs with demonstrated miR ceRNA functions harbor m6A modifications and will be discussed later in the review.

### 3.2 mRNA stabilization

Another function of circRNAs in cancer is the direct stabilization of other mRNAs. It was found that CDR1as expression is increased in NSCLC relative to normal tissues and silencing of CDR1as significantly reduced metastases from the NSCLC subtype, lung squamous cell carcinoma (LUSC) *in vivo*. Overexpression of the protein coding mRNA, CDR1, in CDR1as-silenced cells only partially rescued the metastatic phenotype, suggesting that the function of CDR1as in LUSC is in part due CDR1as-mediated CDR1 mRNA stabilization. No evidence of the widely cited miR-7 ceRNA activity for CDR1as was found in this study (Harrison et al., 2020). This apparent lack of CDR1as miR-7 ceRNA function suggests the same circRNA can possess multiple functions, depending on specific cellular contexts. Secondly, circFOXK2 was shown to be overexpressed in ER + breast cancer and was shown to directly bind the 3′ untranslated region of the cyclin CCND1 mRNA. This interaction facilitates the recruitment of the RBP ELAV1 to stabilize the CCND1 mRNA. Intriguingly, therapeutically targeting circFOXK2 improves tamoxifen response in tamoxifen resistant, ER + breast cancer cells in *in vivo* xenografts (Yi et al., 2025).

### 3.3 IGF2BP family protein interactors

Increasingly prevalent in recent years, circRNAs have been shown to drive cancer progression by interacting directly with proteins. These interactions can prevent the normal activities of the protein or facilitate their degradation/stabilization. Commonly, circRNAs interact with members of the IGF2BP family of RBPs, which are known to have roles in cancer (Ramesh-Kumar and Guil, 2022). CircNDUFB2 directly binds to all three members of the IGF2BP protein family and acts as a scaffold between the IGF2BP proteins and the ubiquitin ligase TRIM25, which marks them for degradation. In addition, circNDUFB2 is recognized by RIG-I and is correlated with the activation of the RIG-I-MAVS signaling pathway to recruit immune cells to *in vivo* tumors. CircNDUFB2 is downregulated in NSCLC, thereby increasing levels of the IGF2BP family and blunting the immune response through deactivation of the RIG-I-MAVS pathway (Li et al., 2021). Secondly, the circRNA CDR1as is downregulated in melanoma models due to H27K3 trimethylation at the transcriptional locus. Silencing of CDR1as in models with high CDR1as expression produced tumorigenic phenotypes *in vitro* and *in vivo*. CDR1as was shown to bind IGF2BP3 as a ceRNA, and subsequent downregulation of CDR1as is suggested to free IGF2BP3 to influence metastatic functions in melanoma (Hanniford et al., 2020). Once more, no evidence of miR-7 ceRNA activity was found for CDR1as in this study, offering additional support for multiple and miR ceRNA-independent functions of circRNAs (Hanniford et al., 2020).

CircRNAs have also been shown to promote the stabilization of mRNAs through their interactions with the IGF2BP family. For example, transforming growth factor beta-induction in colorectal cancer activates and overexpresses circITGB6. CircITGB6 was shown to directly bind IGF2BP3 and promote the stabilization of the PDPN mRNA, and this stabilization is reported to promote the epithelial to mesenchymal transition in *in vivo* experiments (Li K. et al., 2023).

### 3.4 Protein ceRNAs and other protein interactions

CircRNAs also bind other proteins in various cancers, generally functioning as protein ceRNAs. For example, upregulated circFNDC3B directly binds to the TFII-I transcription factor which prevents TFII-I interaction with STAT1 in NSCLC. This TFII-I/STAT1 disruption inhibits the expression of chemokines *in vitro* necessary to attract T cells to the tumor. Overexpression of the mouse ortholog, circFndc3b, in NSCLC cells decreased the effectiveness of anti-PD-1 immunotherapy *in vivo*. Of particular note, circFNDC3B is an ecircRNA that was found to be primarily nuclear, constituting a rare example of an ecircRNA that is retained in the nucleus (Wei et al., 2025). Also in NSCLC, circATF6 was observed to be downregulated in drug tolerant persister cells after osimertinib treatment. Subsequent overexpression of circATF6 results in direct binding to the endoplasmic reticulum (ER) chaperone protein BiP and enhances binding of the circATF6/BiP complex to the ER, which induces a breakdown in the endoplasmic reticulum and calcium accumulation within the cytoplasm *in vitro* (Hu et al., 2025). Moreover, the circRNA circHERC1 contributes to NSCLC growth by acting as a miR ceRNA along with a protein ceRNA. CircHERC1 is upregulated in NSCLC where it directly binds miR-142-3p to increase HMGB1 levels, which activates the MAKP and NF-kB pathways. CircHERC1 also directly binds and sequesters FOXO1 in the cytoplasm, which activates the AKT pathway. Both functions of circHERC1 drive NSCLC cell proliferation *in vitro* and *in vivo* (Cui Y. et al., 2023). Lastly, circ-hnRNPU displays reduced expression in gastric cancer relative to normal tissues. Overexpression of circ-hnRNPU revealed direct binding to the protein NONO and cytoplasmic sequestration of the circ-hnRNPU/NONO pair. This retention in the cytoplasm inhibited NONO transactivation of c-Myc in the nucleus, expression of glycosyltransferases and the hnRNPU parental gene, and reduced tumorigenic phenotypes of gastric cancer cells both *in vitro* and *in vivo* (Li H. et al., 2023).

### 3.5 Peptide production and other epigenetic mechanisms

A subset of circRNAs also possess the ability to produce peptide products. CircFBXW7 generates a peptide product in multiple cancers. In one study, circFBXW7 is downregulated in glioma but harbored a potential open reading frame. The resultant peptide, FBXW7-185aa, was identified and found to directly interact with USP28, which inhibited USP28-induced stabilization of c-Myc *in vitro* (Yang et al., 2018). In another study, circFBXW7 was found to have both miR ceRNA and peptide functions. CircFBXW7 was observed to be downregulated in triple negative breast cancer. Overexpression of circFBXW7 directly binds miR-197–5p, which increases the expression of the parental tumor suppressor gene FBXW7 *in vitro*. Furthermore, the FBXW7-185aa peptide was over expressed and a destabilization of c-Myc was observed *in vitro*. This destabilization was reversed by overexpression USP28 *in vitro* (Ye et al., 2019). These studies provide evidence that the peptide product of circFBXW7 acts through the same pathway in two separate cancers. The prevalence of circRNA-encoded peptides and identification of the peptide’s functions under normal conditions requires further study.

Still other circRNAs possess more unique epigenetic functions in cancer that do not fit into the above mechanisms. In breast cancer where the parental gene is overexpressed, the circRNA FECR1 binds to the parental FLI1 promoter where it recruits and directly binds the demethylase TET1 to induce hypomethylation. Additionally, FECR1 was associated with the methyltransferase DNMT1 promoter where it induces DNMT1 downregulation *in vitro* (Chen et al., 2018). Secondly, an intergenic circRNA circ_0007379 was found to be downregulated in colorectal cancer where it functions as a miR processing scaffold, enabling the production of mature miR-320a. Circ_0007379 enabled miR-320a was then found to downregulate the RUNX1 transcription factor. *In vivo*, overexpression of circ_0007379 in colorectal cancer cell lines increased miR-320a levels and decreased RUNX1 expression. These changes led to decreased colorectal cancer metastasis (Long et al., 2023). Finally, several circRNAs were found to be generated from gene fusion products caused by common chromosomal translocations in leukemias, sarcomas and lung cancer (called f-circRNAs) (Guarnerio et al., 2016). Expression of the f-circRNAs increased cell proliferation *in vitro*, promoted leukemia progression *in vivo*, and enhanced protection of leukemic cells *in vivo,* though the direct mechanism by which the f-circs are operating remains unclear (Guarnerio et al., 2016).

## 4 Regulation of circRNAs via RNA modifications

RNA modifications, particularly m6A, have been demonstrated to play a role in virtually all aspects of circRNA biology. Briefly, in disease-free cellular conditions, m6A modifications deposited by METTL3 and read by YTHDC1 on the circRNA sequence are necessary for efficient backsplicing for a subset of circRNAs (Di Timoteo et al., 2020). These m6A marks, particularly near the circRNA backsplice junction, are also used by cellular sensors to differentiate endogenous circRNAs from exogenous RNAs (Chen Y. G. et al., 2019). Furthermore, the m6A RNA modification and associated readers/writers are also required for translation of a subset of ecircRNAs, though it is uncertain if all ecircRNAs requiring m6A modifications for effective backsplicing are translated based on the same modification (Di Timoteo et al., 2020; Yang et al., 2017). Lastly, m6A modifications on circRNAs can serve as binding sites for the reader YTHDF2, which in turn induce endonucleolytic cleavage of a subset of circRNA through YTHDF2 induced recruitment of HRSP12 and RNase P/MRP (Park et al., 2019).

CircRNAs harboring m6A modifications also have roles the cancer biology through the same mechanisms as non-modified (or undetermined modification status) circRNAs. For example, some m6A modified circRNAs with ceRNA effects drive PI3K/AKT/mTOR pathway activation. CircMDK is upregulated in hepatocellular carcinoma and is transcriptionally stabilized by m6A modifications. CircMDK was shown to act as a ceRNA by directly binding both miR-346 and miR-874-3p, which upregulates their shared target, ATG16L1. Increased ATG16L1 promotes PI3K/AKT/mTOR signaling, and therapeutically targeting circMDK in several *in vivo* models proved efficacious (Du et al., 2022). Secondly, in cisplatin resistant ovarian cancer cell lines, the m6A-stabilized circPLPP4 binds miR-136 and induces upregulation of PIK3R1 to drive PI3K/AKT/mTOR pathway activation. Similarly, targeting circPLPP4 in xenograft models reduced tumor growth (Li et al., 2024). Not all ceRNA effects lead directly to a cell signaling pathway activation. Briefly, circIGF2BP3 contributes to anti-PD-1 immunotherapy evasion in NSCLC by binding miR-328–3p and miR-3173-5p, and thus upregulating PKP3 and stabilizing PD-L1 in tumor cells. Blocking of the circIGF2BP3/PKP3 axis in conjunction with anti-PD-1 immunotherapy in rodent models increased T cell infiltration and activation (Liu et al., 2021).

M6A modified RNAs have also been shown to function through stabilization of mRNAs. For example, m6A marks on circNSUN2 facilitate its export to the cytoplasm through binding to the reader YTHDC1. Increased levels of circNSUN2 drive colorectal cancer metastasis by direct binding to IGF2BP1 and the HMGA2 mRNA, stabilizing the mRNA (Chen R. X. et al., 2019). Additionally, the m6A-marked circRNF13 has been shown to act oncogenically in multiple cancers. CircRNF13 is upregulated in cervical cancer where it stabilizes the CXCL1 mRNA through direct binding. This stabilization leads to radiotherapy resistance of the cervical cancer cells (Shi et al., 2023). CircRNF13 is also upregulated in oral cancer and binds to IGF2BP1, where it enhances IGF2BP1 phase separation and IGF2BP1 stabilization of the ITGB1 mRNA in an m6A dependent manner (Xu et al., 2025).

As an example of a tumor suppressive m6A circRNA, cLMNB1 is downregulated in NSCLC where it was shown to serve as a scaffold for FGFR4 and its ubiquitin ligase CBL. The downregulation of cLMNB1 is facilitated by the m6A levels on the RNA, and a cLMNB1 mutant lacking the m6A modification site prevents Osimertinib resistance in NSCLC (Qian et al., 2025).

The prevalence of m6A modifications and its roles in circRNA function and cancer remained understudied, but the current state of the field has been reviewed in depth elsewhere (Huang et al., 2022; Zhang et al., 2020).

## 5 Engineering circRNAs for effective therapeutic delivery

The majority of available drugs on the market are molecules of low molecular weight, or small molecules. These are synthetic molecules that generally function by binding to proteins to directly inactivate them or by acting as agonists/antagonists on cell surface receptors to activate/inhibit downstream signaling (Southey and Brunavs, 2023). While small molecule therapeutics are highly effective for certain conditions, many genes are widely considered to be “undruggable” as the proteins of interest do not possess the chemical characteristics to interact with small molecules (Hopkins and Groom, 2002).

Another drug class that has undergone extensive optimizations and improvements in recent years are RNA-based therapies. Rather than targeting proteins directly, RNA-based therapies rely on co-opting endogenous cellular mechanisms to post-transcriptionally regulate gene expression by inducing degradation or modifying splicing at the RNA level. Traditional RNA-based therapeutics encompass small interfering RNAs (siRNAs) and antisense oligonucleotides (ASOs), and function through independent, unique mechanisms (Crooke et al., 2018). These RNA-based therapeutics have become viable treatment options and can bypass the “undruggable” protein characteristics by modulating expression pre-translationally.

Finally, an additional RNA-based therapy modality is the direct introduction of mRNA into the cells for translation in the cytoplasm. This modality has gained considerable attention in recent years with the rapid approval of vaccines to combat the SARS-CoV-2 outbreak (Dagan et al., 2021). Specifically in the context of cancer treatments, mRNA-based therapies involve the delivery of synthetic mRNAs to cancer cells to induce expression of antigens that are displayed on the cell membrane to elicit an immune response (Miao et al., 2021). Delivery to the cancer cells is commonly achieved through incorporation of the mRNA into lipid nanoparticles, and several mRNA therapies are in clinical trials (Wadhwa et al., 2020).

While the drug development field is constantly changing and improving, many diseases and conditions remain difficult to treat. Several endogenous properties that make circRNAs biologically unique, such as exonuclease resistance, increased intracellular stability compared with linear mRNAs, and cap-independent translation, has led the field to consider engineered circRNAs as a unique therapeutic modality. Recently, one circRNA therapy (RXRG001) to treat low saliva production and dry mouth in cancer survivors has entered a Phase I clinical trial.

### 5.1 Producing and optimizing circRNAs as therapeutic platforms

Prior to the observation of endogenous circRNA-generated peptides, synthetic circRNAs were engineered to generate proteins in diverse cell types, including human cells (Wang and Wang, 2015). Translation of circRNAs requires an IRES as they do not possess 5′ caps for initiation of translation (Hellen and Sarnow, 2001). The inherent cellular stability of circRNAs relative to linear mRNAs and their ability to be translated marks them as an attractive alternate platform to delivering translatable RNA into cells.

Extensive optimization has gone into producing circularizable linear RNAs capable of driving translation (Petkovic and Muller, 2015). Inclusion of self-splicing group I introns and spacer sequences flanking the desired open reading frame (ORF) to be translated dramatically improved circularization rates and fidelity of the circularized products over complementary sequences in the introns (Cui J. et al., 2023; Meganck et al., 2021; Wesselhoeft et al., 2018). Improvements to IRES sequences used in the constructs boosted translation from the synthetic circRNAs (Chen et al., 2021). Additional optimizations to the 5′ and 3′ untranslated regions and synthetic aptamer regions (to improve recruitment of the translational machinery) similarly boosted translation (Chen R. et al., 2022). CircRNA vectors have even been designed and tested to optimize translation in the intended recipient cell, such as immune cells for immunotherapies (Yu et al., 2025). These improvements to the synthetic, *in vitro* production of circRNAs containing translatable ORFs has clinical promise for a variety of conditions.

### 5.2 Delivery of circRNAs as therapeutic platforms

Once the therapeutic circRNA has been successfully synthesized, it must be delivered to the target tissue *in vivo*. Effective delivery of circRNAs to tissues can be mediated through a variety of methods, including lipid nanoparticles (LNPs), charge-altering releasable transporters (CARTs), or virus-like particles (Gupta et al., 2024; McKinlay et al., 2017). LNPs are a complex of lipids and cholesterol which effectively package the desired cargo, protect the cargo from degradation, and efficiently release the cargo after the LNP has crossed the cellular membrane (Mehta et al., 2023). LNPs have been used to deliver many molecular cargos, such as mRNAs and small interfering RNAs, and have been used as the delivery mechanism in pre-clinical studies to treat cancer (Guo et al., 2021). When complexed with circRNAs, a study found potent anti-tumor responses in three separate models driven by delivery of a synthetic circRNA/LNP formulation (Li et al., 2022). Similarly, an LNP delivered circRNA vaccine designed to present SARS-CoV-2 antigen offered protection from infection of multiple SARS-CoV-2 variants (Qu et al., 2022). As a variation of classic LNPs, CARTs are polymers containing an initiator, a block of lipids, and a polycationic section. The polycationic end allows for the packaging of negatively charged molecules at acidic pHs, and undergoes a conformational shift at physiologic pHs within cells, which releases the complexed cargo and enhances endosomal escape (McKinlay et al., 2017). Improvements to the monomers used in CART synthesis have increased the delivery efficiency of mRNAs into immune cells (Li Z. et al., 2023). Moreover, delivery of optimized circRNA molecules complexed with CARTs into mice effected the desired antigen presentation, stimulated a large T cell response and reduced proliferation of melanoma xenografts (Amaya et al., 2023). While these studies show promise for circRNA therapeutics, all currently remain in the pre-clinical stage.

### 5.3 Current limitations and translational challenges

Numerous advances have been made to the design of traditional RNA-based therapies (siRNAs and ASOs) to increase their cellular stability, increase their binding affinity to the target RNA, reduce immunogenicity, and increase delivery of the therapies to the desired tissues (Crooke et al., 2018; Egli and Manoharan, 2019; Hu et al., 2020; Jadhav et al., 2024; Kaczmarek et al., 2017). Similarly, improvements in cellular stability, translation efficiency, and stimulated immune activation have been made to the mRNA payloads through sequence optimizations, chemical modifications and production techniques (Holtkamp et al., 2006; Kariko et al., 2011; Kariko et al., 2008; Kuhn et al., 2010). CircRNAs need to be synthesized and circularized, and incorporation of many nucleoside modifications presents technical challenges. Additionally, translation of modified nucleosides can prove to be difficult and inefficient, though cap-independent translation enhancers have been identified to improve translation (Wang et al., 2026). The synthesis process of circRNAs is often inefficient or produces unwanted by-products, which makes synthesis challenging of the large amounts required for therapeutic delivery. Additionally, conjugating a targeting ligand to the circRNA for efficient and specific delivery to the target cell is impossible as circRNAs lack a free RNA end for conjugation. For further reference, dedicated reviews on the potential for circRNA therapeutics have been recently published (Chen et al., 2025; Ugwu et al., 2026).

Many studies concerning circRNA mechanisms in cancer suggest that the identified circRNA represents a new therapeutic target. Oncogenic circRNAs represent an attractive target for therapeutics, as expression will be relatively limited to the cancer cells that have re-expressed the circRNA, thus reducing the likelihood of off target effects. However, targeting circRNAs in cancer cells presents its own challenges and limitations. CircRNAs generally do not encode proteins, so effective therapeutic agents will be limited to RNA based therapies designed to cause degradation of the circRNA. Delivery to the desired tissues will present the same challenges as with current, mRNA-targeted RNA therapies. As most actionable circRNAs are produced from the backsplicing of mRNAs, careful sequence selection (i.e., at the backsplice junction) is needed to prevent unintended destruction of the linear mRNA as well. Even with thoughtful sequence selection, RNA-based therapeutics run the risk of reducing the linear mRNA (Lovendorf et al., 2023). Any therapeutics designed for circRNAs will require thorough testing and validation of target specificity.

## 6 Discussion

Although circRNAs have only been recognized as a novel non-coding RNA subset for a few decades, their functional relevance in normal cells and pathophysiologic conditions are becoming increasingly appreciated. CircRNAs are highly conserved and expressed across eukaryotes, display unique and temporally controlled expression patterns, and their biogenesis can occur through a variety of endogenous but non-canonical mechanisms. CircRNAs can be categorized based on their sequence composition, and their cellular location largely determines their function(s). M6A RNA modifications on a subset of circRNAs are required for proper biogenesis, export and degradation. In cancers, circRNAs have been shown to have dysregulated expression patterns consistent with both oncogenic activation and tumor suppressive inhibition. Mechanistically in cancer, circRNAs have been demonstrated to act as ceRNAs for both miRs and proteins, directly and indirectly stabilize mRNAs, act as scaffolding for proteins and generate peptides, all of which yield evidence that altered genetic regulatory mechanisms promote disease states. Furthermore, substantial work has gone into engineering circRNAs to be effective platforms for therapeutic modalities, with the first clinical trial for a circRNA therapeutic currently in progress.

The most widely cited mechanism for circRNA function is acting as a ceRNA or sponge for miRs. The relevance of this miR ceRNA mechanism has been met with skepticism in the field for two primary reasons. Many circRNAs harbor a small number of predicted binding sites for specific miRs, though few circRNAs harbor many binding sites for the specific miR. Additionally, the endogenous expression level of the circRNA is usually far less than the expression level of the miR of interest. The paucity of predicted miR binding sites per circRNA molecule along with dramatic differences in endogenous expression led to the assertion that many circRNAs are incapable of producing a significant miR ceRNA cellular effect (Thomson and Dinger, 2016). In contrast, many recent studies have identified alternate mechanisms of circRNA function, as described. In certain cases, direct circRNA interactions with proteins may be more likely to be biologically relevant than interactions with miRs, depending on the circRNA, miR and miR target gene expression levels. Additionally, direct circRNA regulations of transcription factors, such as MYC, provide a very clear link to cancer phenotypes.

While there are now more resources available to identify and study circRNAs than ever before, there are methodological limitations and considerations to be taken into account. A multitude of tools have been designed to predict binders of circRNAs, for both proteins and miRs (Dudekula et al., 2016). These various circRNA annotation platforms differ in their accuracy, so numerous platforms should be referenced (Table 2). Regardless of the tool used or binder identified, all *in silico* predicted interactions must be validated experimentally. Cellular knockdown of circRNAs (siRNAs, short hairpin RNAs, etc.) must include careful target sequence selection to ensure specific silencing of the circRNA. Primers used for polymerase chain reaction amplification of circRNAs must span the backsplice junction to avoid unintended amplification of the linear mRNA. Proper controls must be used in various circRNA pulldown assays to ensure specific enrichment of the circRNA of interest.

While the circRNA field is progressing at a rapid pace, many aspects of circRNA biology remain understudied. As most circRNAs do not encode for protein, RNA secondary structure may play a vital role in their cellular functions. A few studies relate circRNA structure to function, however thus far none are in the context of cancer (Fischer et al., 2020; Liu et al., 2019). Determining the structure/function relationship of circRNAs will be of vital importance to the field. Similarly, there is evidence for cancer-specific splice isoforms of circRNAs, yet the relative functional consequences of these isoforms are not well characterized thus far (Feng et al., 2019). Proper use of proposed circRNA naming conventions should be used to avoid confusion when beginning study of the many currently unannotated circRNAs (Chen et al., 2023). Finally, the effects of possibly extensive RNA modifications on circRNAs has yet to be fully explored. While certain circRNAs have been found to be marked with modifications such as m6A, it remains unclear whether these modifications are widespread on circRNAs in general. Many circRNAs interact with the m6A reader IG2BP family of RBPs, though it is unknown if all circRNAs that directly interact with this RBP family also harbor m6A modifications (Ramesh-Kumar and Guil, 2022). Studies are lacking or nonexistent that determine the presence of RNA modifications other than m6A on circRNAs, and it is uncertain whether differentially modified circRNAs are biologically active or perform unique cellular functions.

This review connects circRNA dysregulation and cellular mechanisms observed in cancers to normal functions of circRNAs in disease free states. In addition, it emphasizes the importance of multiple potential mechanisms for circRNA function, both in normal conditions and in cancer. Finally, it highlights significant knowledge gaps involving the prevalence and impact of both RNA modifications on circRNAs and understudied classes of circRNAs, while providing possible avenues for future studies. From epigenetic drivers of cancer to potential therapeutics, the future of the circRNA field is full of excitement and opportunities.
